# Probing the mechanism of peptidoglycan amidase activation by FtsEX-EnvC

**DOI:** 10.1128/mbio.02114-25

**Published:** 2025-09-08

**Authors:** Jonathan Cook, Allister Crow

**Affiliations:** 1 School of Life Sciences, University of Warwick 2707, Coventry, United Kingdom; University of Georgia, Athens, Georgia, USA

**Keywords:** structural microbiology, bacterial cell division, bacterial cell envelope, peptidoglycan amidase, type VII ABC transporters, coiled coils, knobs-into-holes packing

## Abstract

The FtsEX-EnvC-AmiA/B system is a key component of the *E. coli* cell division machinery that directs breakage of the peptidoglycan layer during separation of daughter cells. Structural and mechanistic studies have shown that ATP binding by FtsEX in the cytoplasm drives periplasmic conformational changes in EnvC, which lead to the binding and activation of peptidoglycan amidases such as AmiA and AmiB. The FtsEX-EnvC amidase system is highly regulated to prevent cell lysis with at least two separate layers of autoinhibition that must be relieved to initiate peptidoglycan hydrolysis during division. Here, we test the FtsEX-EnvC amidase activation mechanism through site-directed mutagenesis. We identify mutations that disrupt the autoinhibition mechanism of FtsEX-EnvC and an N-terminal deletion variant that prevents activation. Finally, we develop a cysteine locking residue pair that stabilizes the complex in its amidase activating conformation. The reported EnvC variants greatly enhance our understanding of the FtsEX-EnvC autoinhibition mechanism and the conformational changes underpinning amidase activation. Our observations are consistent with the proposed mechanism of amidase activation by large-scale conformational changes in FtsEX-EnvC, allowing recruitment and activation of peptidoglycan amidases.

**IMPORTANCE:**

In *E. coli*, the FtsEX-EnvC system regulates two of the three division-associated amidases that break the peptidoglycan layer during bacterial division. Structural and mechanistic studies have revealed a detailed molecular mechanism for amidase activation in which an ABC transporter and its periplasmic partner reversibly activate periplasmic amidases under direction of the cytoplasmic cell division machinery. This paper explores structural features of EnvC that underpin autoinhibition and the activation mechanism. The FtsEX-EnvC system serves as a powerful example of a Type VII ABC transporter that uses transmembrane conformational changes to drive work in the periplasmic space.

## INTRODUCTION

The peptidoglycan layer is a core component of the bacterial cell envelope that provides both shape and rigidity as well as a point of attachment for the outer membrane (1–5). During cell division, there is a need to break the peptidoglycan layer so that newly formed daughter cells can be separated from one another (6). Breaks in the peptidoglycan layer are also required to expose new connection points from which additional peptidoglycans can be added during bacterial elongation (3). In *E. coli*, two division-associated peptidoglycan amidases (AmiA and AmiB) are each located in the periplasm and are regulated by a Type VII ABC transporter complex: FtsEX-EnvC (7–9). FtsEX-EnvC is recruited to the Z-ring early in division (10, 11) and regulates the activity of AmiA and AmiB through transmembrane conformational changes driven by FtsEX (8, 9, 12, 13).

*E. coli* strains lacking FtsE, FtsX, or EnvC typically form long chains of cells that are connected by the unbroken peptidoglycan layer (13). These strains are susceptible to antibiotics and detergents that would not normally cross the outer membrane barrier and have reduced viability on low salt media, indicating cell envelope defects (8, 11, 14–16). Such phenotypes are best understood as the result of peptidoglycan defects arising from the absence of proper amidase activation. However, amidase variants have also been reported with promiscuous activity that produce partial cell lysis (9, 17, 18). Both inhibition of amidase activation and promiscuous activation of amidases have therefore been seen as potential routes for developing useful antimicrobials targeting cell envelope integrity and bacterial cell division.

**Fig 1:**
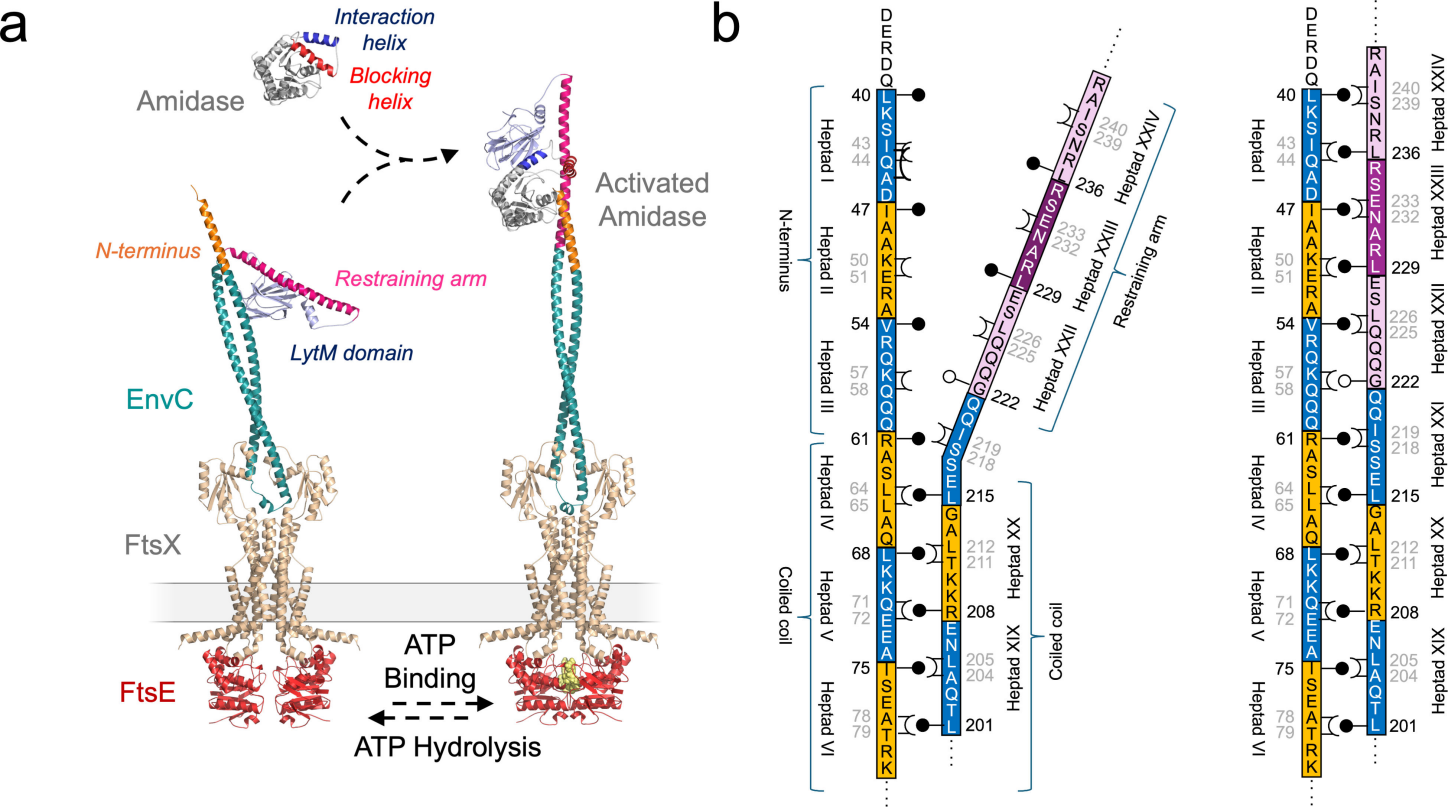
Mechanism of amidase activation by *E. coli* FtsEX-EnvC. (**a**) ATP binding to FtsE in the cytoplasm causes a transmembrane conformational change to propagate through the FtsEX-EnvC complex, leading to the binding and activation of a peptidoglycan amidase. ATP hydrolysis resets the system, causing the release (and deactivation) of the amidase as the FtsEX-EnvC complex returns to its inactive resting state. Models shown are hypothetical (i.e., modeled rather than experimentally determined) but consistent with structures that have been described previously (8, 23, 24). (**b**) Diagram showing the proposed knobs-into-holes packing between the EnvC N-terminus and the restraining arm. Knobs indicated as black pins, and holes are indicated with crowns. G222 is indicated by a white-filled pin to emphasize the absence of a sidechain in this knob position.

A molecular mechanism for the *E. coli* FtsEX-EnvC-AmiA/B system has been developed and is depicted in Fig. 1a. In their resting states, periplasmic amidases such as AmiA and AmiB are unable to bind and hydrolyze peptidoglycans because an internal autoinhibitory helix blocks access to their respective active sites (9, 18, 19). Activation of AmiA or AmiB requires a conformational change in the amidase that displaces the amidase autoinhibitory helix and allows rearrangement of residues around the active site zinc ion (9). The conformational changes in AmiA or AmiB are induced by the binding of a cognate murein hydrolase activator (EnvC) (8, 9, 20–22). A crystal structure of a periplasmic amidase bound to the EnvC LytM domain has shown the interaction of amidase with a specific binding groove in the EnvC LytM domain (9). Binding of the amidase’s “interaction helix” causes displacement of the amidase’s “blocking” helix, exposing the active site to the peptidoglycan layer (9). EnvC is also subject to its own autoinhibition mechanism (8) and is bound to the periplasmic domains of a Type VII ABC transporter (FtsEX) (8, 12, 13). In its resting state, the EnvC amidase binding site (located within its C-terminal LytM domain) is blocked by a long helix termed the restraining arm, which must be displaced to allow the binding of amidases (8). As depicted in the figure, ATP binding in the cytoplasm drives a transmembrane conformational change that propagates through both FtsEX and the EnvC coiled coil domain, displacing its restraining arm and facilitating the binding of amidases (Fig. 1a). ATP hydrolysis returns the system to the inactive resting state after a period of activation. The FtsEX-EnvC-AmiA/B system represents a remarkable example of conformational change with multiple levels of autoinhibition that protect the cell from improper peptidoglycan hydrolase activity.

Activation of EnvC relies on the elongation of its coiled coil domain and involves multiple heptad repeats in the protein N-terminus and the restraining arm that are predicted to be paired up in the active conformation but parted in the resting state (8) (Fig. 1b). Formation of the extended coiled coil during activation is supported by the identification of co-evolving residue pairs between the restraining arm and N-terminus and identification of residues that would support classical knobs-into-holes packing (8, 25) (Fig. 1b). Knobs-into-holes describes the interlocking of anti-parallel helices via 7-residue “heptad” repeats in which the residue at the first position (the “knob”) locks between the fourth and fifth positions (the “hole”) of an opposing heptad on a second helix. The EnvC conformational change mechanism is broadly supported by observations from various partial structures of the FtsEX-EnvC-AmiA/B complex (8, 9, 18, 22), cryoEM data (23, 24, 26–28), and hypothetical models (8, 9); however, the importance of the reversible formation of an extended coiled coil remains to be tested.

Here, we examine the importance of three structural features within EnvC: (1) the restraining arm responsible for autoinhibition (2); a potential molecular hinge between the restraining arm and the coiled coil domain; (3) the N-terminus of EnvC, which supports the formation of the “active” amidase-recruiting conformation through the formation of an extended coiled-coil. Using a combination of *in vitro* and *in vivo* work, we identify mutations that disrupt the autoinhibition mechanism and test the importance of the proposed reversible knobs-into-holes packing that underpins the proposed conformational changes in EnvC. These data enhance our understanding of the EnvC autoinhibition mechanism and the structural features underpinning amidase activation by the FtsEX-EnvC-AmiA/B system.

## RESULTS

### Mutations in the restraining arm disrupt the autoinhibition mechanism of EnvC

**Fig 2:**
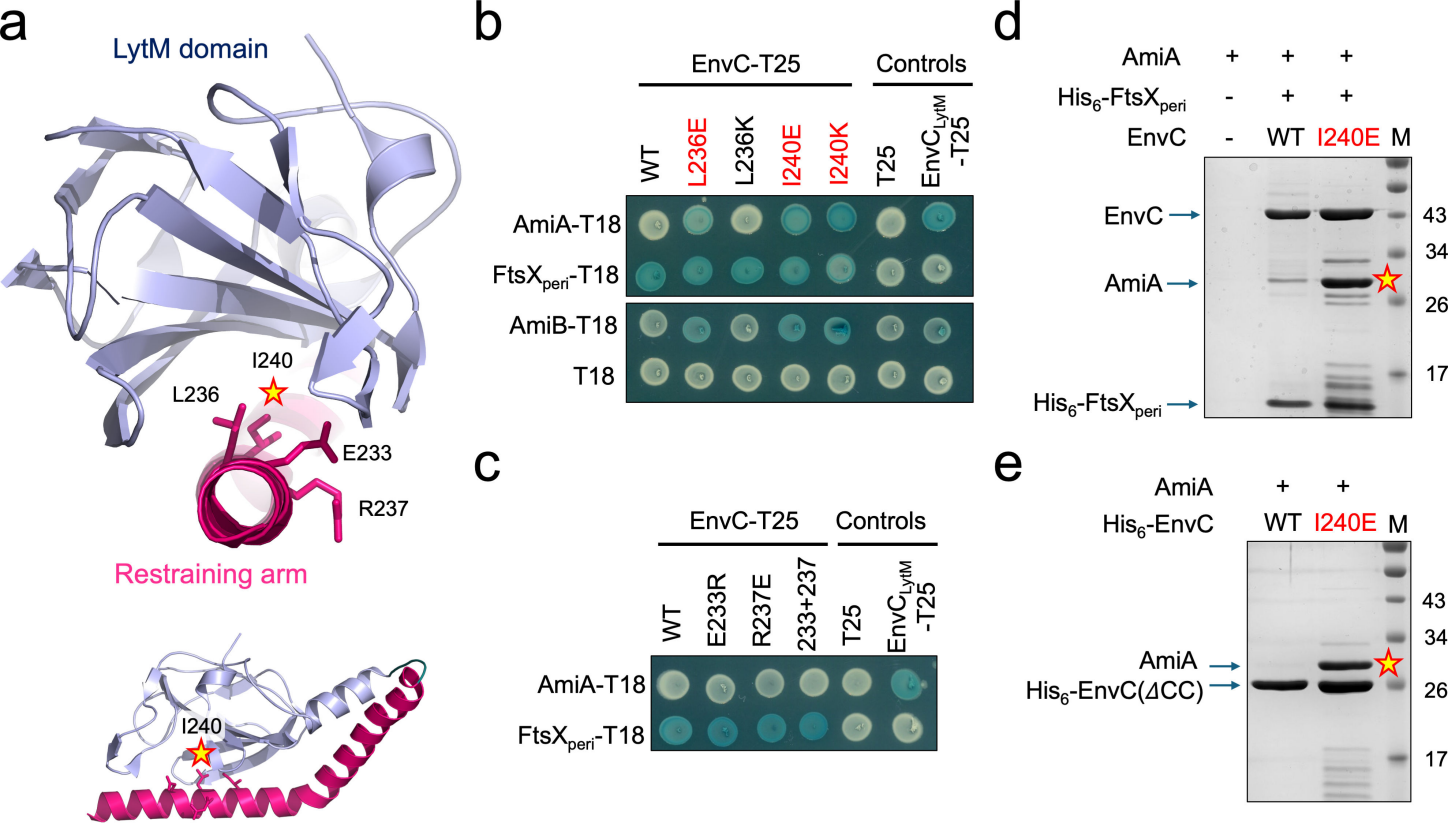
Mutations in the EnvC restraining arm overcome autoinhibition and promote amidase binding. (**a**) Structure of EnvC with focus on the restraining arm. Stars indicate the locations of the autoinhibition-breaking mutations identified herein. (**b**) Bacterial two-hybrid assay testing the interaction between EnvC variants and the two division-associated periplasmic amidases (AmiA and AmiB). The interaction with the FtsX periplasmic domain forms an additional control to show that all full-length EnvC proteins are expressed and folded. Red lettering indicates EnvC mutations that overcome autoinhibition and allow promiscuous amidase binding. T18 and T25 indicate fusions to the small and large parts of the split-CyaA protein used for detecting protein-protein interactions. (**c**) Bacterial two-hybrid assay probing the interaction between EnvC variants carrying restraining arm mutations that do not break autoinhibition. (**d**) Comparative co-purification of AmiA with a soluble complex composed of EnvC and the FtsX periplasmic domain. A pair of lysates containing over-expressed AmiA and the EnvC-FtsX periplasmic domain complex were mixed and used as input for purification using the Ni-IMAC resin. Only the FtsX periplasmic domain is His-tagged. A star indicates a dense AmiA band arising from copurification with I240E EnvC. (**e**) Comparative co-purification of AmiA with truncated forms of EnvC that lack the N-terminal coiled coil domain but retain both the LytM domain and restraining arm. A star indicates a dense AmiA band that only co-purifies with the I240E variant.

The EnvC restraining arm is a key structural element that prevents the binding and activation of peptidoglycan amidases (8). In its resting state, the restraining arm of EnvC blocks access to the amidase binding groove located in the LytM domain, preventing amidase activation. However, in the activated conformation, the restraining arm is displaced, allowing amidases to bind (8, 9). A pair of hydrophobic residues (Leu236 and Ile240) each appear to be important for maintaining the restraining arm in the autoinhibited state as they lock inside the hydrophobic groove that would otherwise accommodate the amidase (Fig. 2a).

We predicted that substituting hydrophobic residues for charged residues might release the restraining arm and overcome the EnvC autoinhibition mechanism. To test this, we used a bacterial two-hybrid experiment monitoring the interaction between EnvC and its cognate amidases, AmiA and AmiB, and made either lysine or glutamate mutations in Leu236 and Ile240. These bacterial two-hybrid experiments were conducted in the cytoplasm using proteins representative of their mature periplasmic forms. In line with previous observations (8), neither AmiA nor AmiB interact with the wild-type EnvC (consistent with its autoinhibition by the restraining arm), while the isolated EnvC LytM domain, for which the restraining arm is absent, interacts with both (Fig. 2b). In keeping with our predictions, we found that L236E, I240E, and I240K EnvC variants all interact with both AmiA and AmiB (Fig. 2b).

Having established that the hydrophobic residues at positions 236 and 240 are needed for autoinhibition, we next considered two nearby charged residues (Glu233 and Arg237) that are located on the restraining arm but face away from the LytM domain. These residues form useful counter-predictions as they are not expected to be important for maintaining autoinhibition but are positioned close to Leu236 and Ile240. Consistent with their structural context, Glu233Arg or Arg237Glu substitutions do not disrupt autoinhibition, and these EnvC variants remain unable to bind AmiA or AmiB in the bacterial two-hybrid assay—even though both variants interact with the periplasmic domain of FtsX, suggesting they are competently expressed and folded (Fig. 2c). Taken together, these data show that L236E, I240E, and I240K mutations on the restraining arm all disrupt the EnvC autoinhibition mechanism, allowing binding of peptidoglycan amidases.

### Characterization of the I240E autoinhibition-breaking mutation

Having identified mutations that break the EnvC autoinhibition mechanism in the bacterial two-hybrid assay, we selected our most effective mutation, I240E, for further characterization *in vitro*. We have previously shown that EnvC can be co-purified with the truncated periplasmic domains of FtsX and solved a crystal structure of this complex (8). However, the truncated FtsX-EnvC complex does not readily bind to either AmiA or AmiB due to the EnvC autoinhibition mechanism. We therefore tested whether introduction of the I240E mutation disrupts autoinhibition and allows co-purification of peptidoglycan amidases with the truncated FtsX-EnvC complex (Fig. 2d). We first mixed cell lysates containing the His-tagged soluble FtsX-EnvC complex and AmiA (without a His-tag) and immobilized the resultant complexes on Ni-IMAC resin. After washing, the remaining proteins were eluted from the resin and analyzed by SDS-PAGE to identify potential amidase-activator complexes. As a control, we also performed the experiment using the AmiA lysate without any FtsX-EnvC present. As expected, untagged AmiA does not bind the empty IMAC resin and has only a weak interaction with the wild-type FtsX-EnvC complex. However, the introduction of the I240E mutation in the truncated FtsX-EnvC complex produced a much stronger interaction with AmiA consistent with disruption of autoinhibition and unimpeded binding of the amidase (Fig. 2d).

To further probe the I240E mutation, we explored amidase binding to a minimal protein construct that is composed of only the EnvC LytM domain and restraining arm (Fig. 2e). The EnvC(ΔCC) construct lacks the N-terminal coiled coil domain but is expected to remain autoinhibited due to the presence of the restraining arm. We found no evidence of AmiA binding to the wild-type EnvC(ΔCC) construct, but strong binding to its I240E variant (Fig. 2e). These results demonstrate the importance of Ile240 in maintaining EnvC in its inactive conformation and the ability of the I204E mutation to overcome autoinhibition.

### The I240E mutation causes growth defects when expressed in the periplasm

**Fig 3:**
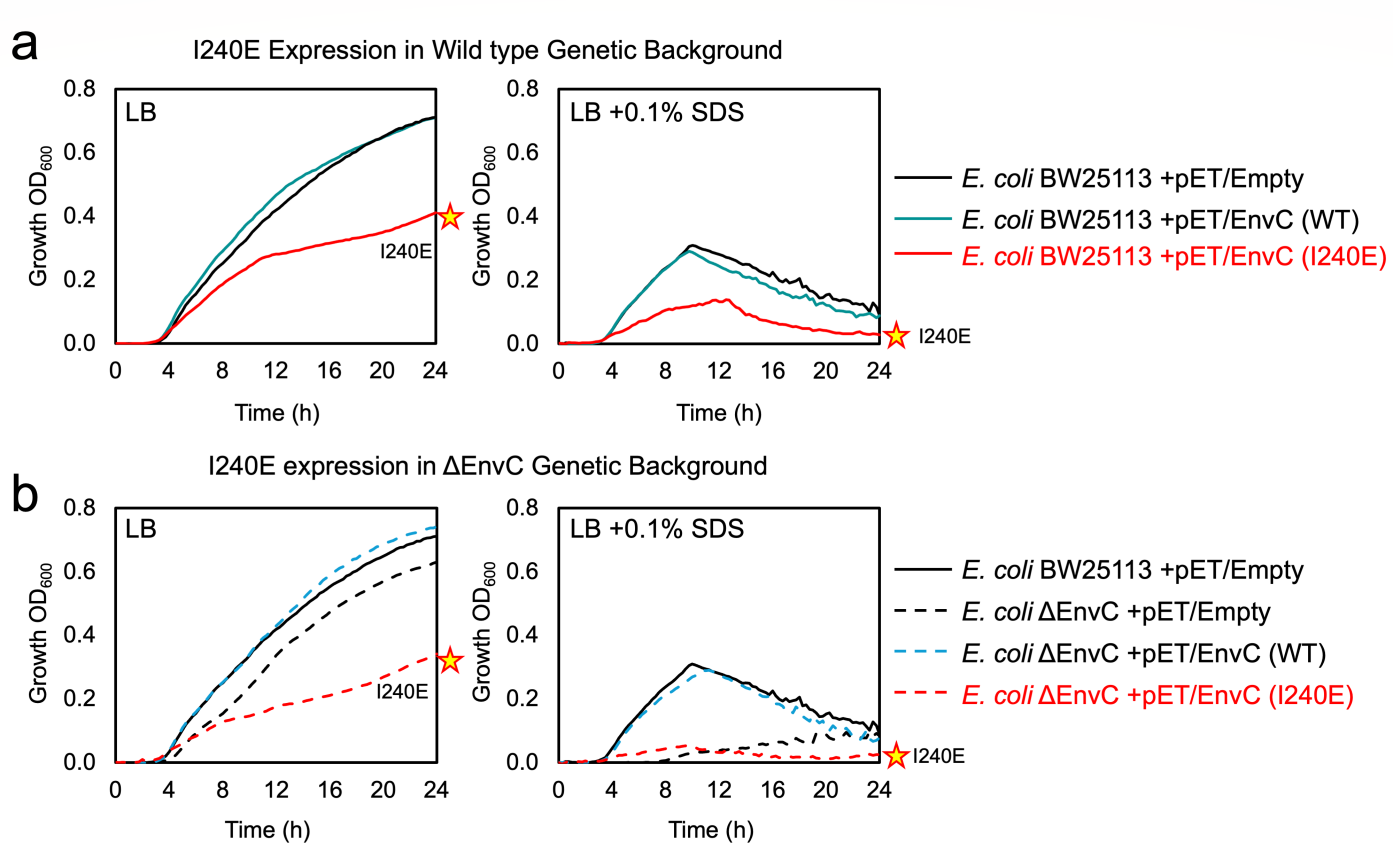
The EnvC I240E variant shows growth defects and SDS sensitivity. (**a**) Growth curves for *E. coli* BW25113 in LB broth (left) and LB broth supplemented with SDS (right). Strains each harbor a different pET22-based plasmid expressing the indicated EnvC variant. A strain carrying the empty vector is used as a control. A star indicates the impaired growth curve due to expression of the EnvC I240E variant. (**b**) A similar set of growth curves using an isogenic strain that lacks the chromosomal *envc* gene. All growth curves are the mean of multiple repeats.

Having shown that the I240E mutation in the restraining arm causes EnvC to readily bind amidases in both the bacterial two-hybrid and co-purification assays, we next tested whether this mutation has a disruptive effect *in vivo*. Our prediction was that the I240E variant should promiscuously bind and activate amidases, causing outer membrane defects and impairing bacterial growth. To test, we expressed the I240E EnvC variant in the periplasm of *E. coli* and monitored bacterial growth in LB broth. Periplasmic expression of the wild-type EnvC protein had no effect on bacterial growth compared to the negative control; however, expression of the I240E EnvC variant significantly impaired growth (Fig. 3a *left*). When challenged with broth containing SDS detergent, growth was further reduced for the strain expressing the EnvC I240E variant consistent with outer membrane defects (Fig. 3a *right*). We also repeated these experiments using an EnvC knockout strain (Fig. 3b). Strains lacking the chromosomal copy of *envc* exhibit modest growth impairment and severe detergent sensitivity, with both phenotypes rescuable by expression of the wild-type EnvC from a plasmid (Fig. 3b). However, when the I240E variant was introduced into the *envc* knockout strain, the growth defect was intensified (Fig. 3b *left*) and the cells remained detergent-sensitive (Fig. 3b *right*). Taken together, these data are consistent with our characterization of I240E as a mutation that disrupts the EnvC autoinhibition mechanism and facilitates promiscuous binding and activation of peptidoglycan amidases in the periplasm.

### Mutations targeting a proposed molecular hinge in EnvC

**Fig 4:**
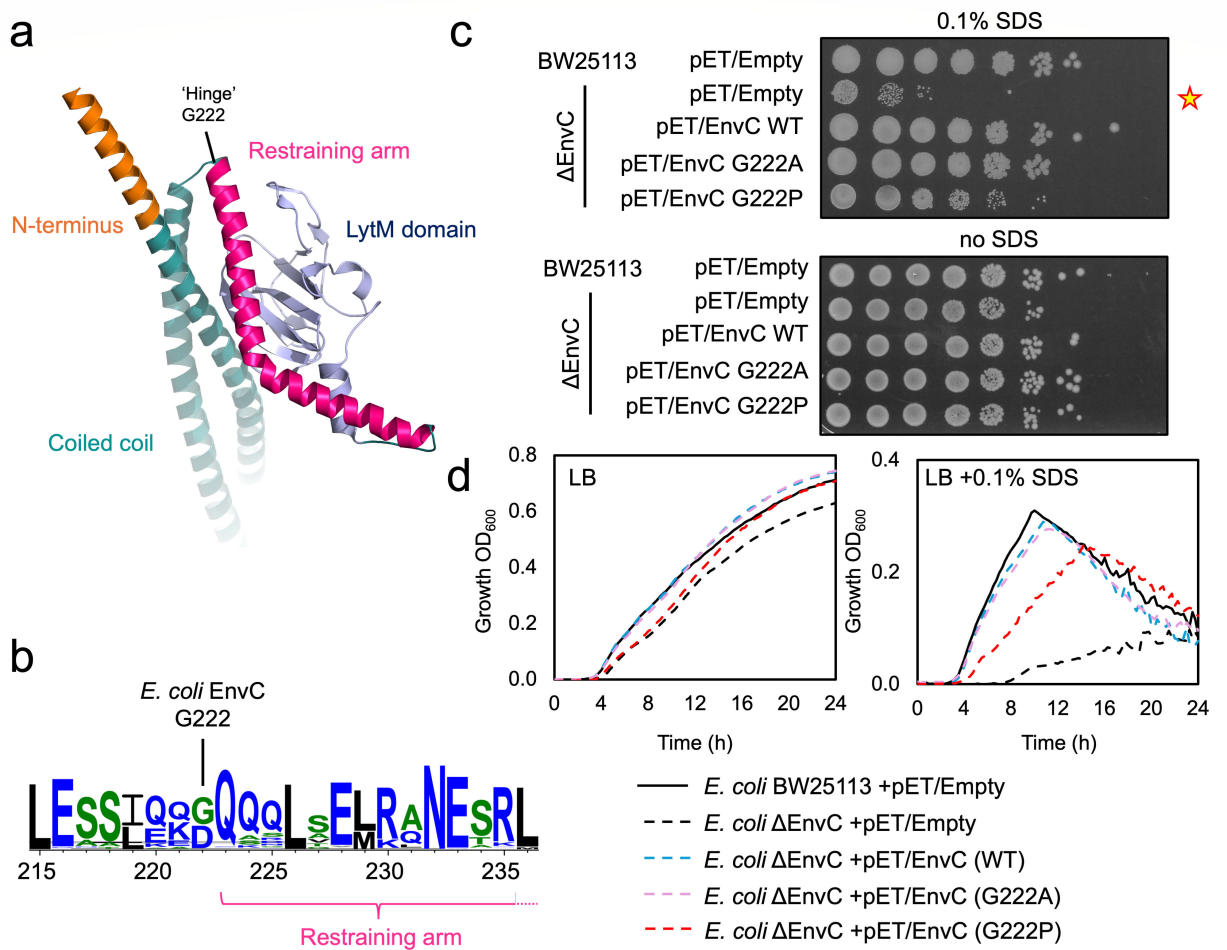
Mutations in the molecular hinge of EnvC. (**a**) Structural figure showing the EnvC hinge. (**b**) Weblogo representations of residue conservation at the EnvC hinge. (**c**) Detergent sensitivity assays for strains dependent on EnvC hinge variants. Tenfold serial dilutions of the indicated cultures are spotted from left to right on an LB agar plate containing 0.1% SDS. Stars indicate reduced detergent viability for strains that lack EnvC or are solely dependent on an EnvC Gly222Pro variant. (**d**) Growth curves for indicated strains grown in LB or LB supplemented with SDS detergent. All growth curves are the mean of at least three repeats.

Structures of EnvC in its autoinhibited conformation (8) suggest the presence of a “hinge” region connecting the N-terminal coiled coil domain with the restraining arm (Fig. 4a). During activation, the hinge is expected to transition between amidase-activating and autoinhibited conformations. In the autoinhibited state, the hinge is bent as per the crystal structure of EnvC, but in the activated state, it is predicted that these residues adopt a continuous helical conformation that links the coiled coil with the restraining arm. Straightening of the hinge thus allows knobs-into-holes packing between heptads in the restraining arm and the N-terminus. We identified a glycine residue (G222) that might provide intrinsic flexibility to the hinge (Fig. 4a and b). Gly222 is present among many enterobacterial EnvC proteins and is located at what is expected to be a knob position in heptad XXII (see Fig. 1b).

The additional flexibility and the absence of a conventional “knob”-like side chain led us to further investigate the importance of Gly222. We first made alanine and proline substitutions and tested their ability to complement the cell envelope defects of an EnvC-deficient strain. Alanine has a high propensity to form alpha helical secondary structures (29); thus, we predicted the Gly222Ala variant may have a tendency toward activation. On the other hand, proline, due to its side chain ring structure, lacks the backbone N-H group needed to form an alpha helical structure, which might potentially block activation. *E. coli* cells lacking EnvC have reduced viability on SDS agar due to cell envelope defects. Complementation with either the wild-type protein or Gly222Ala EnvC restored viability on SDS agar to the wild-type levels, while the Gly222Pro variant only partly complemented EnvC deficiency (Fig. 4c). Similar results were found for growth in LB broth containing detergent (Fig. 4d), suggesting that EnvC Gly222 variants have a modestly impaired function compared to the wild-type.

While the effect of each Gly222 variant was broadly in line with our predictions, the magnitude of these phenotypic differences was small—especially in comparison to other mutations established in the FtsEX-EnvC-AmiA system. We therefore conclude that Gly222 may have some minor mechanistic importance as part of the EnvC hinge, but it is not an essential residue for the FtsEX-EnvC activation mechanism.

### The EnvC N-terminus is necessary for amidase activation

**Fig 5:**
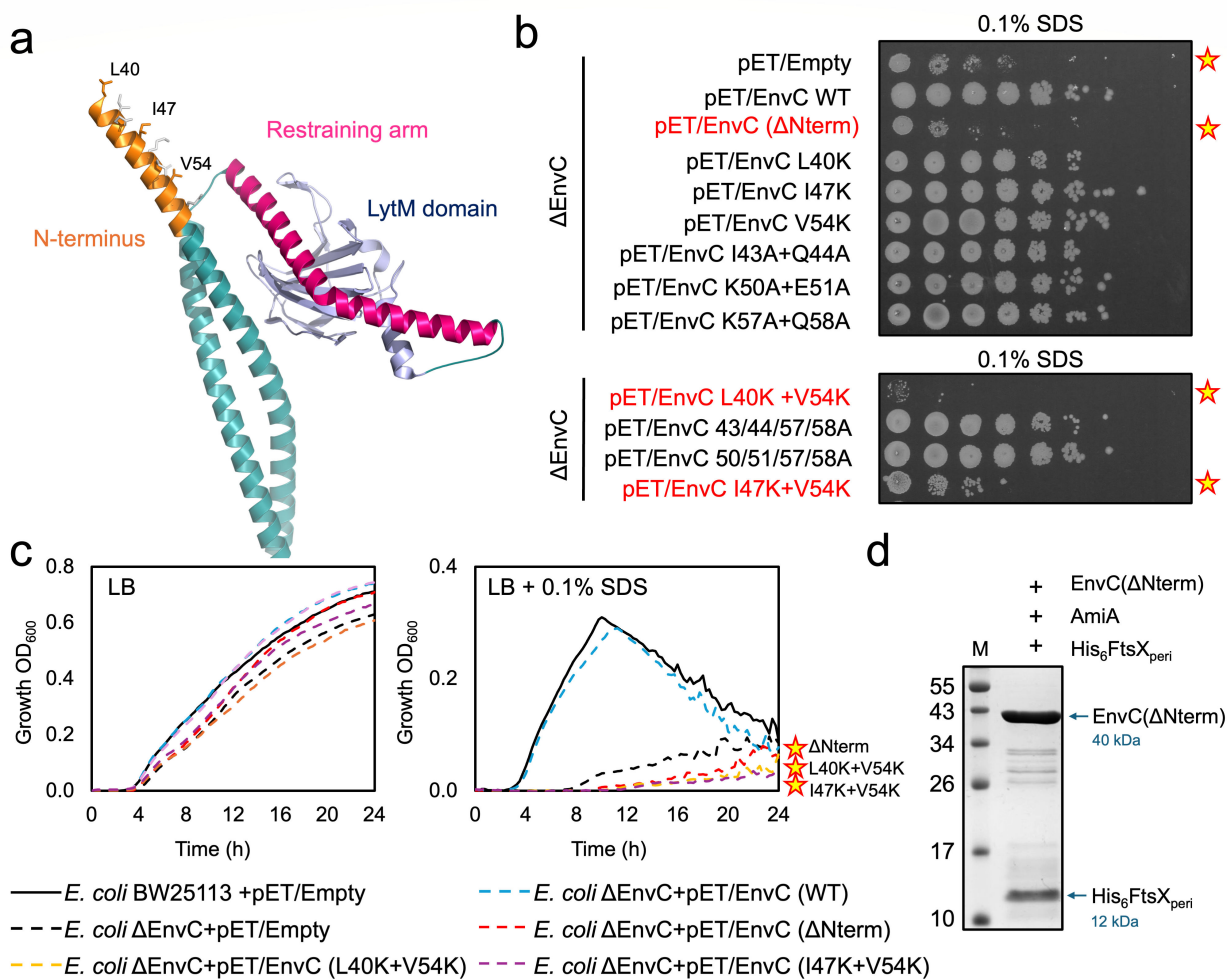
The EnvC N-terminus is required for activation of FtsEX-EnvC. (**a**) Close-up view of the EnvC N-terminus. (**b**) Detergent viability assays for strains dependent on the plasmid-based expression of indicated EnvC variants. Stars indicate reduced viability on SDS agar due to mutations in the EnvC N-terminus. (**c**) Growth curves in LB broth containing the SDS detergent. A star indicates an SDS-sensitive strain that is dependent on EnvC lacking the N-terminus. (**d**) Purification of the EnvC(ΔNterm) variant in complex with the FtsX periplasmic domain in the presence of AmiA. All growth curves are the mean of multiple repeats.

The N-terminus of EnvC consists of an exposed portion of alpha helix that contains three heptad repeats. In the autoinhibited conformation, these three heptads are unpaired and face the solvent (Fig. 5a), but in the activated conformation, these heptads have been predicted to pair with heptads in the restraining arm (8). The reversible interaction between the EnvC N-terminus and the restraining arm is a core prediction of the currently understood mechanism of conformational change that underpins the recruitment and activation of amidases by FtsEX-EnvC, but, to our knowledge, this has not been tested by mutagenesis.

To experimentally test the importance of the EnvC N-terminus, we made mutations in the N-terminal heptads and explored each variant’s ability to complement the SDS sensitivity phenotype of an *envc* knockout strain (Fig. 5b). Amino acid substitutions were chosen to disrupt the predicted knobs-into-holes packing expected to underpin the interaction of the N-terminus with the restraining arm. We also generated an EnvC construct that lacks the N-terminus completely—EnvC(ΔNterm). When expressed in the bacterial periplasm via a pET22-based vector, the wild-type EnvC protein completely rescues the SDS sensitivity of the *envc* knockout strain. However, cells expressing EnvC without the N-terminus remain sensitive to SDS, suggesting this feature is essential for EnvC function (Fig. 5b).

We note that periplasmic expression of EnvC variants from a pET-based vector in BW25113 relies on recognition of the plasmid T4 promoter by the housekeeping RNA polymerase and consequently does not involve protein over-expression. Consistently, SDS-PAGE analysis of the periplasmic fraction shows no evidence of overexpressed proteins (Fig. S1a). However, periplasmic expression of EnvC variants was confirmed using the same plasmids in C43(DE3), which carries a T4 polymerase and produces a larger amount of protein—sufficient to visualize by SDS-PAGE (Fig. S1b). In the case of EnvC(ΔNterm), which is smaller than the other constructs, SDS-PAGE analysis was complicated by the presence of an overlapping background band that is also present in the empty vector control. We therefore confirmed periplasmic expression of EnvC(ΔNterm) separately by generating a C-terminal His-tagged form and purifying it directly from the periplasmic fraction using Ni-IMAC alongside a similarly constructed wild-type protein and other key variants (Fig. S1c and d).

To further test the hypothesized importance of individual heptads, we assessed point mutations in the EnvC N-terminus (Fig. 5b). Individual disruption of the “knob” residues from each of the three N-terminal heptads (L40K, I47K, and V54K) did not oblate EnvC function, and neither did individual disruption of any of the three residue pairs (I43A + Q44A, K50A + E51A, and K57A + Q58A) that form the “holes” within each heptad. However, two EnvC variants lacking pairs of knob residues (L40K + V54K and I47K + V54K) were unable to complement *envC* deficiency, lending credence to the hypothesized knobs-into-holes packing that underpins the activation of EnvC. These variants were confirmed to be periplasmically expressed using SDS-PAGE after expression in *E. coli* C43(DE3) (Fig. S1b). We also tested whether the EnvC N-terminal deletion variant could complement *envc* deficiency on the knockout strain in LB broth containing SDS (Fig. 5c). Expression of wild-type EnvC completely complements the SDS-sensitive phenotype, while the protein lacking the N-terminus was unable to do so. To control for the possibility that disruption of the N-terminus might simply impair the stability of EnvC, we made an EnvC expression construct in which the N-terminus was deleted and co-expressed it alongside the periplasmic domain of FtsX. The EnvC(ΔNterm)-FtsX periplasmic domain complex purifies well, showing that deletion of the N-terminus does not disrupt the expression and folding of EnvC and that it remains capable of binding to FtsX (Fig. 5d). The EnvC(ΔNterm)-FtsX periplasmic domain construct also remains unable to bind AmiA, showing that removal of the N-terminus does not disrupt the EnvC autoinhibition mechanism (Fig. 5d). Taken together, these data support the hypothesized importance of the EnvC N-terminal heptad repeats in the activation mechanism of FtsEX-EnvC.

### Cysteine-locking of EnvC in its amidase-activating conformation

**Fig 6:**
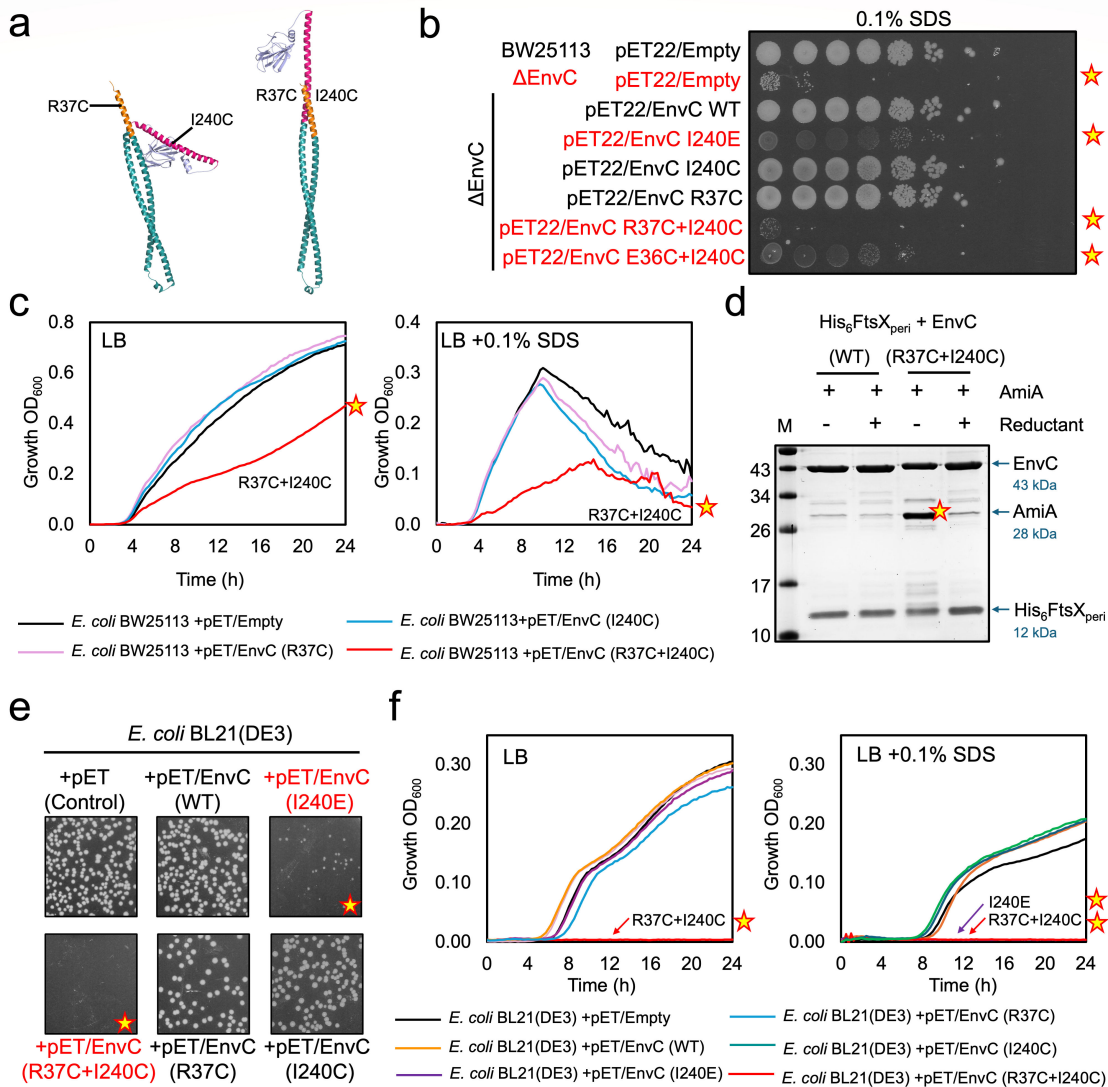
Disulfide locking of EnvC in an active conformation using engineered cysteine residues in the N-terminus and restraining arm. (**a**) Structural figure indicating the proposed crosslink position in EnvC. (**b**) Detergent sensitivity assay for strains grown on SDS agar. A tenfold serial dilution for each culture was spotted on the agar in series from left to right starting at OD_600_ 1. Stars indicate detergent-sensitive strains. (**c**) Growth curves in LB or LB broth containing the SDS detergent. Star indicates growth of a strain expressing the EnvC(R37C + I240C) cross-linkable variant. (**d**) Co-purifications of the cross-linkable EnvC(R37C + I240C)-FtsX periplasmic domain complex in the presence of AmiA. Only the FtsX periplasmic domain is His-tagged. AmiA co-purifies with the cross-linked variant under oxidizing conditions but not in the presence of reductant (TCEP). (**e**) Transformation of BL21(DE3) with pET vectors encoding EnvC variants. Stars indicate toxic variants with low transformation efficiency. (**f**) Growth curves for *E. coli* BL21(DE3) expressing indicated EnvC variants on LB (*left*) or LB supplemented with SDS (*right*). Stars indicate strains that did not grow.

To further test the hypothesized knobs-into-holes packing expected in the activated FtsEX-EnvC complex, we attempted to trap the activated complex using a cysteine locking experiment. Based on modeling of the activated complex as a perfect coiled coil, we introduced pairs of cysteine residues into the EnvC N-terminus and restraining arm with the anticipation that these would form disulfide bonds when expressed in the periplasmic space. While we initially predicted that cysteine locking would be most effective between cognate “knob” and “hole” residues, our *in silico* modeling suggested that opposing “hole” residues may in fact be better suited for optimal disulfide geometry (Fig. 6a). After some experimentation, we identified a cysteine pair variant (R37C + I240C) that appears to fit the bill. When expressed in the periplasm of a strain lacking the chromosomal copy of *envc*, the R37C + I240C EnvC variant renders cells sensitive to SDS, even though the corresponding single-cysteine variants (R37C, I240C) are unaffected (Fig. 6b). A similar, albeit weaker SDS-sensitive phenotype is also observed for cells that retain their chromosomal *envc*, suggesting a dominant effect (Fig. S2). A second cys-locking pair, E36C + I240C, was also identified (Fig. 6b; Fig. S2) although this was not analyzed further.

Subsequent experiments in LB broth showed that expression of R37C + I240C EnvC not only fails to complement the cell envelope defect of the *envc* knockout but also actively impairs the growth of wild-type cells consistent with improper activation of amidases (Fig. 6c). In fact, a wild-type *E. coli* strain expressing the R37C + I240C EnvC variant shows a greater growth defect in LB than a strain that lacks EnvC. These data suggest that R37C + I240C very likely forms a disulfide bond and can activate amidases in the periplasmic space.

It is noteworthy that the I240C mutation used for disulfide locking the restraining arm to R37C in the N-terminus is the same position as the I240E mutation used to disrupt the autoinhibition mechanism. This makes the comparison of the I240E and I240C single mutants particularly insightful as they each have distinct properties. Complementation of the EnvC-deficient strain with the I240C variant completely rescues growth to the level of the wild-type EnvC, while the I240E mutation exacerbates growth defects. Furthermore, the expression of I240C in wild-type cells has only minimal effects on growth, while expression of I240E causes growth defects. When grown on LB agar containing SDS, the EnvC-deficient strain expressing I240E EnvC produces translucent colonies that are noticeably smaller than wild-type, while the I240C variant rescues the EnvC deficiency and appears completely normal (Fig. 6b). These comparisons demonstrate that I240C alone does not disrupt autoinhibition, while I240E does. The different behavior of the two I240 mutations is explained by the nature of the substitutions—a cysteine residue is easily accommodated within the amidase-binding groove of the EnvC LytM domain, while the charged glutamate (I240E) is incompatible with the available space and hydrophobic character of the groove.

To further test the hypothesis that the cysteine-locked variant binds and activates amidases, we purified R37C + I240C EnvC in complex with the FtsX periplasmic domain and tested its ability to bind AmiA compared to the wild-type in both the presence and absence of a disulfide-breaking reductant (Fig. 6d). We found that the soluble EnvC-FtsX periplasmic domain complex has minimal affinity for AmiA (consistent with autoinhibition) and is unaffected by the presence or absence of reductant (Fig. 6d *left*). However, the R37C + I240C variant binds AmiA strongly in the absence of the reductant, but not at all when the reductant is present (Fig. 6d *right*). Reversibility of the amidase binding function of the cysteine-locking variant due to the addition of a disulfide-breaking reductant indicates that the R37C + I240C variant only binds the amidase when a disulfide bond is present between the N-terminus and the restraining arm. The experiment also confirms that amidase binding cannot be explained by the I240C mutation dislodging the restraining arm as the R37C + I240C variant does not bind the amidase under reducing conditions. The combined data from *in vivo* and *in vitro* are consistent with R37C + I204C forming an intramolecular disulfide bond that locks the protein in an activated conformation, allowing amidase recruitment and activation.

### Phase-contrast microscopy shows a weak chaining phenotype for EnvC knockouts and partial lysis for activation-prone variants

*E. coli* strains lacking FtsEX, EnvC, or lacking combinations of peptidoglycan amidases (e.g., AmiA, AmiB, and AmiC) have each been reported to show “chaining” phenotypes consistent with failure to effectively break the peptidoglycan layer during division. We therefore examined the morphology of cells expressing I240E and the R37C + I240C, alongside appropriate controls, in both wild-type and ΔEnvC backgrounds. Wild-type *E. coli* BW25133 cells grow as short rods and are unaffected by expression of wild-type EnvC from a plasmid (Fig. S3a and b). In contrast, BW25113 ΔEnvC cells form multi-cell chains that can be restored to wild-type morphology by plasmid-borne EnvC (Fig. S3g and h). Surprisingly, expression of EnvC I240E did not noticeably affect the morphology of wild-type cells (Fig. S3c), although it did induce partial lysis in BW25113 ΔEnvC (detected as cellular debris) and alleviated the chaining phenotype in surviving cells (Fig. S3i). This mixture of lysis and complementation may indicate that the EnvC I240E variant is close to a tipping point between “normal” activity and hyperactive toxicity. For the R37C + I240C EnvC variant, we observed plentiful debris (consistent with partial lysis) and elongated cells in both the wild-type and ΔEnvC cells (Fig. S3d). No lysis was observed in either of the single cysteine controls (EnvC I240C or EnvC R37C) (Fig. S3e, k, f and l) consistent with the R37C + I240C lytic phenotype being dependent on the formation of a disulfide bond. We note that the morphological phenotypes observed here are all relatively subtle—especially in comparison to the distinctive chaining phenotypes observed for strains lacking FtsEX or strains lacking multiple amidases (e.g., ΔAmiABC) which form much longer chains than ΔEnvC. Nonetheless, the microscopy data are broadly consistent with our observations from viability assays and growth curves and suggest I240E and R37C + I240C are activation-prone EnvC variants.

### The cysteine-locking EnvC variant and I240E variant are toxic when expressed in *E. coli* BL21(DE3)

Heretofore, we have only considered the expression of EnvC variants in *E. coli* BW25113, which lacks the DE3 lysogen necessary for high levels of protein expression from pET-based vectors. We therefore tested the expression of I240E and the R37C + I240C EnvC variants in *E. coli* BL21(DE3), which is optimized for higher protein expression. Our prediction was that overexpression of activation-prone EnvC variants should produce strong phenotypes that would be simpler to characterize. We first noted that transformation of BL21(DE3) with plasmids encoding R37C + I240C and I240E EnvC variants was highly inefficient (Fig. 6e), leading us to suspect these variants are toxic. The low transformation efficiency of plasmids encoding EnvC I240E and R37C + I240C variants contrasts with those encoding the wild-type EnvC and the empty vector control, which are readily transformed into BL21(DE3) (Fig. 6e). Fortunately, all BL21(DE3) transformants could be propagated in LB supplemented with glucose to repress leaky expression. We, therefore, measured growth curves in LB broth to assess the toxicity of each EnvC variant once glucose was removed. In BL21(DE3), the R37C + I240C EnvC variant was completely inviable on LB containing IPTG, while the wild-type, both single-cysteine variants, and the empty vector control grew well (Fig. 6f *left*). The I240E variant was also viable, although it did not grow as well as the wild-type. When challenged with SDS detergent, the toxic phenotype was much clearer, with both I240E and the R37C + I240C variants rendered inviable in the presence of IPTG (Fig. 6f *right*). These data support the proposal that I240E and the R37C + I240C EnvC variants are constitutively active in the periplasm and cause bacterial death via unregulated amidase activation.

## DISCUSSION

FtsEX-EnvC has a well-understood molecular mechanism of amidase activation built on many years of research from numerous groups (Fig. 1). Here, we have experimentally tested key aspects of the proposed molecular mechanism using site-directed mutagenesis. We show that the EnvC autoinhibition mechanism relies on hydrophobic interactions between the restraining arm and the amidase-binding groove of the LytM domain that can be disrupted by single-amino acid mutations (Fig. 2). These mutations overcome the EnvC autoinhibition mechanism and facilitate inauthentic recruitment of both AmiA and AmiB. Residue Ile240 appears especially important with charged residue substitutions, producing growth defects when expressed in *E. coli* (Fig. 3). We also probed the role of Gly222 within the EnvC “hinge” that is expected to underpin the conformational change between autoinhibited and amidase-recruiting conformations (Fig. 4). Proline substitution of Gly222 slightly hinders EnvC function, but the glycine does not appear to be essential. We also challenged the hypothesized knobs-into-holes packing that is suspected to occur between the restraining arm and the protein N-terminus during activation of EnvC (Fig. 5). Deletion of the N-terminus, or disruption of the heptad repeats within it, blocks EnvC’s amidase activation function. Finally, we show that introducing a disulfide bond between the restraining arm and the EnvC N-terminus renders EnvC prone to continuous activation, facilitating the unimpeded binding of amidases (Fig. 6). Collectively, these experiments provide ample support for the currently understood mechanism of amidase activation by FtsEX-EnvC and underline the importance of both the EnvC N-terminus and restraining arm for regulation of periplasmic peptidoglycan amidases.

The mechanism by which FtsEX-EnvC couples ATP binding and hydrolysis to the activation of periplasmic amidases is now understood in extensive molecular detail and supported by numerous experiments and structural observations. However, there remain aspects of the wider FtsEX-EnvC mechanism that need to be further explored. Most pressingly, the current understanding of amidase regulation is that amidases are controlled by a series of nested autoinhibition mechanisms that are ultimately relieved by the binding and hydrolysis of ATP by FtsEX; however, it remains unclear what, if anything, regulates ATP binding and hydrolysis on the cytoplasmic side of the membrane. Similarly, while it is understood that FtsEX-EnvC is recruited to the division site by interactions with the Z-ring (11), the molecular nature of these interactions remains to be deduced. Future experiments are needed to establish precisely how FtsEX-EnvC interacts with other components of the cell division machinery and how these interactions regulate the ATP binding and hydrolysis cycle underpinning amidase activation in the periplasm.

Looking beyond *E. coli*, there are various mechanisms of FtsEX-EnvC-like systems identified across the bacterial kingdom. This is especially true of FtsEX-EnvC-like systems such as FtsEX-RipC and FtsEX-PcsB, where the EnvC-like components are enzymatically active and therefore do not recruit a separate amidase to begin peptidoglycan hydrolysis (12, 26, 30–32). Recent structures of the *Mycobacterium tuberculosis* FtsEX-RipC system suggest that RipC lies flat in the inactive conformation and is straightened upon activation by ATP-driven FtsEX mechanotransmission (26). It remains unclear whether these systems use autoinhibition mechanisms to regulate their enzymatic domains or whether it is solely the localization of their enzymatic domains relative to the peptidoglycan layer that determines their processivity.

One practical use for building a better understanding of the FtsEX-EnvC-amidase system is to develop inhibitors that block cell division or cause uncontrolled activation of peptidoglycan amidases. There are now several mutations in the FtsEX-EnvC-AmiA/B system that emulate the effect of such hypothetical molecules. For example, mutations in the ATP-binding site that interfere with nucleotide binding (K41A) or hydrolysis (E163Q) or mutations that weaken the interaction between FtsEX and EnvC (e.g., F152E in FtsX), each block the amidase activation mechanism and have phenotypes that mimic cells lacking the genes encoding FtsE, FtsX, or EnvC (8). Deletion or disruption of the EnvC N-terminus has a similar effect, preventing the activation of amidases in a genetic background that lacks the wild-type EnvC. Conversely, mutations in EnvC that relieve the autoinhibition mechanism (such as the EnvC I240E mutation and disulfide-locking R37C + I240C mutations reported here) promote promiscuous activation of peptidoglycan amidases, causing growth and cell envelope defects—even when expressed in the wild-type genetic background. Other (dissimilar) “dominant” variants have been reported previously—for example, the expression of amidases lacking their internal autoinhibition helix (or bearing point mutations that loosen its grip) can cause bacterial lysis (9, 18, 19). However, amidase variants and both the I240E and R37C + I240C variants reported here are not as effective in producing defects as the isolated EnvC LytM domain (8, 22) (which can freely bind and activate multiple amidases in the periplasm without regulation by FtsEX), perhaps suggesting there are additional layers of amidase regulation that are not yet fully appreciated. Notably, however, none of these variants seem to render the bacterium completely inviable, suggesting that cells possess robust compensatory mechanisms that can, at least partially, mitigate the effect of promiscuous amidase activation. It, therefore, remains unclear whether targeting FtsEX-EnvC or the amidases represents a viable strategy for drug development.

In summary, we investigated key structural features of EnvC that are proposed to underpin an ATP-driven conformational change in the FtsEX-EnvC complex that regulates the binding and activation of periplasmic peptidoglycan amidases during cell division. We found extensive support for the reversible knobs-into-holes conformational change in EnvC and identified both the protein N-terminus and Ile240 within the restraining arm as key mechanistic residues involved in both autoinhibition and amidase activation by FtsEX-EnvC.

## MATERIALS AND METHODS

### DNA constructs

A table describing the DNA constructs used in this report appears in the Supplemental Data (Table S1). For cloning, PCR typically used Q5-based DNA polymerase and primers engineered to include restriction sites at their 5′ end. Cloning then used standard restriction enzymes for cutting DNA and T4 DNA ligase for assembly. EnvC constructs lacking their N-terminus were generated by PCR starting at base 111 (coding for Arg55) and ligated into an appropriate pET vector (e.g., pET22 for periplasmic expression or pET-Duet for cytoplasmic co-expression with the FtsX periplasmic domain). Point mutations were introduced using QuikChange Site-directed Mutagenesis and subcloned into target vectors using restriction-based cloning methods. All DNA constructs were confirmed by DNA sequencing (Genewiz).

### Bacterial two-hybrid assays

Protein-protein interactions were monitored using the BACTH system (33). Assays were performed in *E. coli* BTH101 cells transformed with compatible plasmid pairs. Bacterial two-hybrid interactions were tested in the cytoplasm using protein fusions representative of the mature proteins found in the periplasm after cleavage of their signal sequences. For example, AmiA comprises residues 35–448, while EnvC uses residues 35–419. Further information on the sequences and plasmids used is given in Table S1. After transformation, cell cultures were grown overnight at 30°C in LB supplemented with 50 µg/mL ampicillin and 25 µg/mL kanamycin. Cultures were then spotted onto LB agar plates containing 40 µg/mL X-gal, 0.5 mM IPTG, 50 µg/mL ampicillin, and 25 µg/mL kanamycin. Agar plates were incubated at 20°C for 3 days and imaged using an Epson document scanner.

### Protein co-purification assays

*E. coli* C43(DE3) transformed with pET-based plasmids encoding AmiA or EnvC variants with/without a periplasmic domain of FtsX were grown at 30°C in 2YT media. Protein expression was induced at OD 0.6 using 1 mM IPTG and progressed overnight at 30°C. Cells were pelleted by centrifugation (10 min, 6,000 × *g*), resuspended in Buffer A (26 mM Imidazole, 300 mM NaCl, 50 mM HEPES pH 7.2) and then broken by sonication (seven 30 s pulses using a Bandelin Sonopuls instrument). Lysates were clarified by centrifugation (20 min, 30,000 × *g*) and mixed as required for each experiment. Mixed lysates were then incubated with Bio-Rad Ni-IMAC resin overnight at 4°C with continuous agitation via a tube roller. Each protein-bound IMAC resin was transferred to an empty PD10 column and extensively washed with the same buffer used for resuspension. Remaining proteins were then eluted from the resin with a high imidazole buffer (Buffer B: 250 mM Imidazole, 300 mM NaCl 50 mM HEPES pH 7.5) and analyzed by SDS-PAGE.

For cysteine locking experiments, purifications were performed similarly with modifications to account for the use of TCEP. Briefly, the EnvC-FtsX periplasmic domain complex (and equivalent R37C + I240C variant) were each prepared as a cleared cell lysate and split into two equal volumes. Then, 5 mM TCEP was added to one fraction and omitted from the other. After 1 h agitation at 4°C, both volumes were mixed with a cleared lysate containing untagged AmiA (with or without TCEP). Each volume was then incubated with Bio-Rad Ni-IMAC resin overnight at 4°C. Bound proteins were washed with 20 column volumes of wash buffer (35 mM imidazole, 300 mM NaCl, 50 mM HEPES pH 7.2) supplemented with 5 mM TCEP as required. A final wash was performed using wash buffer without TCEP before eluting with Buffer B. Eluted proteins were separated using SDS-PAGE.

### EnvC complementation experiments

Complementation experiments used *E. coli* BW25113 or an otherwise isogenic *envC* knockout strain from the Keio collection. BW25113 and *Δenvc* strains were transformed by electroporation with pET22-based vectors designed to express either wild-type or variant EnvC proteins in the periplasm. An empty pET22 plasmid was used as a negative control. Cell envelope integrity of each strain was assessed using sensitivity to SDS detergent on both solid agar and in LB broth. Briefly, cells were grown in LB supplemented with 50 µg/mL ampicillin and adjusted to an OD of 1 (at 600 nm) before serial dilution in fresh LB (10-fold each time). Each dilution series was then spotted onto solid LB agar plates (1 mM IPTG and 50 µg/mL ampicillin), made with, or without, 0.1% SDS and grown overnight at 37°C before imaging via an Epson document scanner. For liquid broth experiments, OD 1 cultures were diluted 1,000-fold and a 20 µL volume used to seed 200 µL cultures of LB with 50 µg/mL ampicillin and 1 mM IPTG (or additionally supplemented with 0.1% SDS) in 96-well plates. Growth curves were then measured at 37°C using the OD at 600 nm as a proxy for bacterial proliferation. Measurements were taken every 15 minutes using a shaking plate reader (Thermo Multiskan Sky). All growth curves are the average of three technical repeats. The individual repeats chosen for display are representative samples from 3 to 9 biological repeats (Fig. S4).

### EnvC variant overexpression in BL21(DE3)

Chemically competent *E. coli* BL21(DE3) were transformed with 20 ng plasmid (pJC7514, pJC7515, pJC7517, pJC7518, or empty vector) and plated on LB agar containing 1% glucose. Cells were then grown to OD 1 in LB broth containing 50 µg/ml ampicillin, diluted 100-fold, and then seeded into 200 µL of fresh LB containing ampicillin and 1 mM IPTG (with or without 0.1% wt/vol SDS) in 96-well plates. Growth curves were constructed from OD measurements at 600 nm wavelength taken at 15 min intervals for 24 hours. Cultures were grown at 37°C and shaken before each measurement in the plate reader. For the transformation efficiency demonstration shown in Fig. 6e, BL21(DE3) was transformed with indicated plasmids and plated on LB agar containing 50 µg/mL ampicillin but without additional glucose.

### Periplasmic fractionation and small-scale Ni-IMAC purification of periplasm-expressed EnvC variants

Periplasmic fractions of *E. coli* BW25113 or C43(DE3) were generated by osmotic shock using Tris-sucrose. Briefly, cells were transformed with appropriate plasmids and grown for 2.5 h in 20 mL LB at 37°C. Expression was induced by addition of 1 mM IPTG for 3.5 h at 30°C. Cells were pelleted by centrifugation (4,000 *g*, 8 min) and resuspended in 600 µL Tris-sucrose solution (300 mM Tris-HCl pH 8.0, 20% (wt/vol) sucrose, 1 mM EDTA, 0.5 mM MgCl_2_). Cells were kept at room temperature for 10 min before being pelleted and resuspended in 300 µL ice-cold 1 mM Tris-HCl pH 7.5. After 10 min, the samples were centrifuged to remove the cytoplasmic and membrane fractions (17,000 *g*, 15 min, 4°C) and the supernatant recovered as the periplasmic fraction. To compare the expression of EnvC variants, periplasmic extracts were analyzed by SDS-PAGE. For BW25113, expression from pET vectors did not yield enough protein to reliably identify EnvC. However, expression in C43(DE3) produced clear bands that could be identified by both their apparent molecular weight and absence of a corresponding band in fractions produced from an empty vector control.

For small-scale purification of EnvC variants from the periplasm, we used expression plasmids encoding C-terminal His-tag variants that are otherwise identical to the pET-based plasmids used in our complementation studies. *E. coli* C43(DE3) cells were transformed with the appropriate plasmid (pJC7514, pJC7515, pJC7517, and pJC7518), grown in LB with 2% glucose at 37°C for 2.5 h. Cells were pelleted and resuspended in LB (without glucose) and induced by addition of 1 mM IPTG. After 3.5 h at 30°C, cells were harvested by centrifugation, and the periplasmic fraction was recovered using the Tris-sucrose method recounted above. A control was also performed with the empty vector (pET22a). Periplasmic fractions were incubated with Ni-IMAC resin for 18 h and the protein-bound resin transferred to microspin purification columns. In each subsequent step, the liquid was removed by benchtop centrifugation (13,000 rpm, 10 min, 10°C). The protein-bound resin was washed three times with Buffer A (26 mM Imidazole, 300 mM NaCl, 50 mM HEPES pH 7.2) and eluted with Buffer B (250 mM imidazole, 300 mM NaCl 50 mM HEPES pH 7.5). Purified proteins were then analyzed using SDS-PAGE. We also performed the same analysis using whole cells (broken by sonication and cleared by centrifugation) in place of the periplasmic fraction as input for the purification. For some SDS-PAGE samples, it was necessary to concentrate using acetone precipitation; two volumes of acetone were added to purified periplasmic extracts and placed at −20°C for 30 min. Precipitated proteins were collected at 17,000 × *g*, 15 min, 4°C. Pellets were air-dried, resuspended in Laemmli sample buffer, and analyzed by SDS-PAGE.

### Microscopy

*E. coli* BW25113 cells were transformed with indicated pET plasmids and grown in LB containing 50 µg/mL ampicillin for 2 h at 37°C, followed by induction with 1 mM IPTG for a further 2 h. Cells were then deposited on an LB agar pad under a glass coverslip and imaged using phase contrast microscopy at 100 x magnification.

### Structure analysis and bioinformatics

Homologous sequences for EnvC were identified using the KEGG database (34). Multiple sequence alignments were constructed with Kalign (35) and visualized using Weblogo (36). Structural figures were generated using Pymol (37).

## SUPPLEMENTAL MATERIAL

Supplemental Information mbio.02114-25-s0001.docx Table S1 and Figures S1 to S4.

ASM does not own the copyrights to Supplemental Material that may be linked to, or accessed through, an article. The authors have granted ASM a non-exclusive, world-wide license to publish the Supplemental Material files. Please contact the corresponding author directly for reuse.
