# Supplementary material for: Probing the mechanism of peptidoglycan amidase activation by FtsEX-EnvC
Source: mBio. 2025 Sep 8;16(10):e02114-25. doi: 10.1128/mbio.02114-25 (PMC12505981; doi:10.1128/mbio.02114-25)
Supplement: Supplemental Information — Table S1 and Figures S1 to S4. [file mbio.02114-25-s0001.docx]

Supplemental Information

**Probing the mechanism of peptidoglycan amidase activation by FtsEX-EnvC**

Jonathan Cook, Allister Crow

**Table S1: Plasmids used in this study**

| **Plasmid** | **Vector** | **Contents** | **Label in Figure** | **Figure** |
| --- | --- | --- | --- | --- |
| pJC6 192 | pUT18C | FtsX (110-209) -T18  *FtsX periplasmic domain for B2H* | FtsX_peri_-T18 | 2b,2c |
| pTB1 011 | pUT18C | AmiA (35-289) -T18  *AmiA for B2H* | AmiA-T18 | 2b,2c |
| pJC6 238 | pUT18C | AmiB (23-445) -T18  *AmiB for B2H* | AmiB-T18 | 2b |
| pJC6 124 | pKNT25 | EnvC (35-419) -T25  *EnvC for B2H* | EnvC-T25 | 2b,2c |
| pJC6 257 | pKNT25 | EnvC (278-419) -T25  *EnvC lytM domain for B2H* | EnvC_LytM_-T25 | 2b,2c |
| pJC6 743 | pKNT25 | EnvC (35-419) -T25 (L236E) | EnvC-T25 L236E | 2b |
| pJC6 745 | pKNT25 | EnvC (35-419) -T25 (L236K) | EnvC-T25 L236K | 2b |
| pJC6 747 | pKNT25 | EnvC (35-419) -T25 (I240E) | EnvC-T25 I240E | 2b |
| pJC6 751 | pKNT25 | EnvC (35-419) -T25 (I240K) | EnvC-T25 I240K | 2b |
| pJC6 753 | pKNT25 | EnvC (35-419) -T25 (E233R) | EnvC-T25 E233R | 2c |
| pJC6 757 | pKNT25 | EnvC (35-419) -T25 (R237E) | EnvC-T25 R237E | 2c |
| pJC6 761 | pKNT25 | EnvC (35-419) -T25 (E233R+R237E) | EnvC-T25 233+237 | 2c |
| - | pUT18C | Empty vector control for B2H  T18 fragment | T18 | 2b,2c |
| - | pKNT25 | Empty vector control for B2H  T25 fragment | T25 | 2b,2c |
| - | pET22 | Empty vector control for complementation experiments | pET/Empty | 3a,3b,4c,4d |
| pJC6 260 | pET22 | EnvC (35-419)  *EnvC with pelB signal for periplasmic expression* | pET/EnvC (WT) | 3a,3b,4c,4d,5b,5c,6b,6e,6f |
| pJC6 957 | pET22 | EnvC (35-419) (I240E)  *EnvC with pelB signal for periplasmic expression* | pET/EnvC (I240E) | 3a,3b,4c,6b,6e,6f |
| pJC6 960 | pET22 | EnvC (55-419)  *EnvC without mature N-terminus, with pelB signal for periplasmic expression* | pET/EnvC (ΔNterm) | 5b,5c |
| pJC6 986 | pET22 | EnvC (35-419) (R37C)  *with pelB signal for periplasmic expression* | pET/EnvC R37C | 6b,6c,6e,6f |
| pJC6 994 | pET22 | EnvC (35-419) (I240C)  *with pelB signal for periplasmic expression* | pET/EnvC I240C | 6b,6c,6e,6f |
| pJC6 987 | pET22 | EnvC (35-419) (R37C+I240C)  *with pelB signal for periplasmic expression* | pET/EnvC R37C+I240C | 6b,6c,6e,6f |
| pJC6 988 | pET22 | EnvC (35-419) (E36C+I240C)  *with pelB signal for periplasmic expression* | pET/EnvC E36C+I240C | 6b |
| pJC7 166 | pET22 | EnvC (35-419) (G222A)  *Hinge mutant for periplasmic expression* | pET/EnvC G222A | 4c,4d |
| pJC7 202 | pET22 | EnvC (35-419) (L40K) | pET/EnvC L40K | 5b |
| pJC7 204 | pET22 | EnvC (35-419) (I43A+Q44A) | pET/EnvC I43A+Q44A | 5b |
| pJC7 234 | pET22 | EnvC (35-419) (I47K) | pET/EnvC I47K | 5b |
| pJC7 236 | pET22 | EnvC (35-419) (V54K) | pET/EnvC V54K | 5b |
| pJC7 238 | pET22 | EnvC (35-419) (K57A+Q58A) | pET/EnvC K57A+Q58A | 5b |
| pJC7 244 | pET22 | EnvC (35-419) (K50A+E51A) | pET/EnvC K50A+E51A | 5b |
| pJC7 275 | pET22 | EnvC (35-419) (L40K+V54K) | pET/EnvC L40K+V54K | 5b, 5d |
| pJC7 277 | pET22 | EnvC (35-419) (I43A+Q44A+K57A+Q58A) | pET/EnvC 43/44/57/58 | 5b |
| pJC7 285 | pET22 | EnvC (35-419) (K50A+E51A+K57A+Q58A) | pET/EnvC 50/51/57/58 | 5b |
| pJC7 291 | pET22 | EnvC (35-419) (I47K+V54K) | pET/EnvC I47K+V54K | 5b, 5d |
| pJC7 322 | pET22 | EnvC (35-419) (G222P)  *Hinge mutant for periplasmic expression* | pET/EnvC G222P | 4c,4d |
| pJC6 763 | pET21 | AmiA (35-289)  *No His-tag, for in vitro protein binding experiments* | AmiA | 2d,2e,5d,6d |
| pJC6 474 | pETDuet1 | EnvC (222-419)  *N-terminal His tag, no secretion signal, no coiled coil domain, for in vitro protein experiments* | His_6_-EnvC(ΔCC) WT | 2e |
| pJC6 775 | pETDuet1 | EnvC (222-419) (I240E)  *N-terminal His-tag, no secretion signal, no coiled coil domain, for in vitro protein experiments* | His_6_-EnvC(ΔCC) I240E | 2e |
| pJC6 120 | pETDuet1 | FtsX (110-209) + EnvC (35-419)  *Co-expression system for the EnvC-FtsX periplasmic domain complex. His-tag on c-terminus of FtsX periplasmic domain. For in vitro protein experiments* | His_6_-FtsXperi and EnvC | 2d,6d |
| pJC6 766 | pETDuet1 | FtsX peri (110-209) + EnvC (35-419) I240E  *Co-expression system for the EnvC-FtsX periplasmic domain complex. His-tag on c-terminus of FtsX periplasmic domain. For in vitro protein experiments* | His_6_-FtsXperi and EnvC I240E | 2d |
| pJC6 968 | pETDuet1 | FtsX peri (110-209) + EnvC (R37C+I240C)  *Co-expression system for the EnvC-FtsX periplasmic domain complex. His-tag on c-terminus of FtsX periplasmic domain. For in vitro protein experiments* | His_6_-FtsXperi and EnvC (R37C+I240C) | 6d |
| pJC7 148 | pETDuet1 | FtsX peri (110-209) + EnvC (55-419)  *Co-expression system for the EnvC(*ΔNterm*) -FtsX periplasmic domain complex. EnvC is missing the mature N-terminus. His-tag on c-terminus of FtsX periplasmic domain. For in vitro protein experiments* | His_6_-FtsXperi and EnvC (ΔNterm) | 5d |
| pJC7 514 | pET22 | *For periplasmic expression of EnvC variants. Uses the pelB signal sequence and encodes C-terminal His tag.* | EnvC-His_6_ (WT) | S1 |
| pJC7 515 | pET22 | *For periplasmic expression of EnvC (I240E). Uses the pelB signal sequence and encodes C-terminal His tag.* | EnvC-His_6_ (I240E) | S1 |
| pJC7 517 | pET22 | *For periplasmic expression of EnvC* (R37C+I240C)*. Uses the pelB signal sequence and encodes C-terminal His tag.* | EnvC-His_6_ (R37C+I240C) | S1 |
| pJC7 518 | pET22 | *For periplasmic expression of EnvC (*ΔNterm*). Uses the pelB signal sequence and encodes C-terminal His tag.* | EnvC-His_6_ (ΔNterm) | S1 |


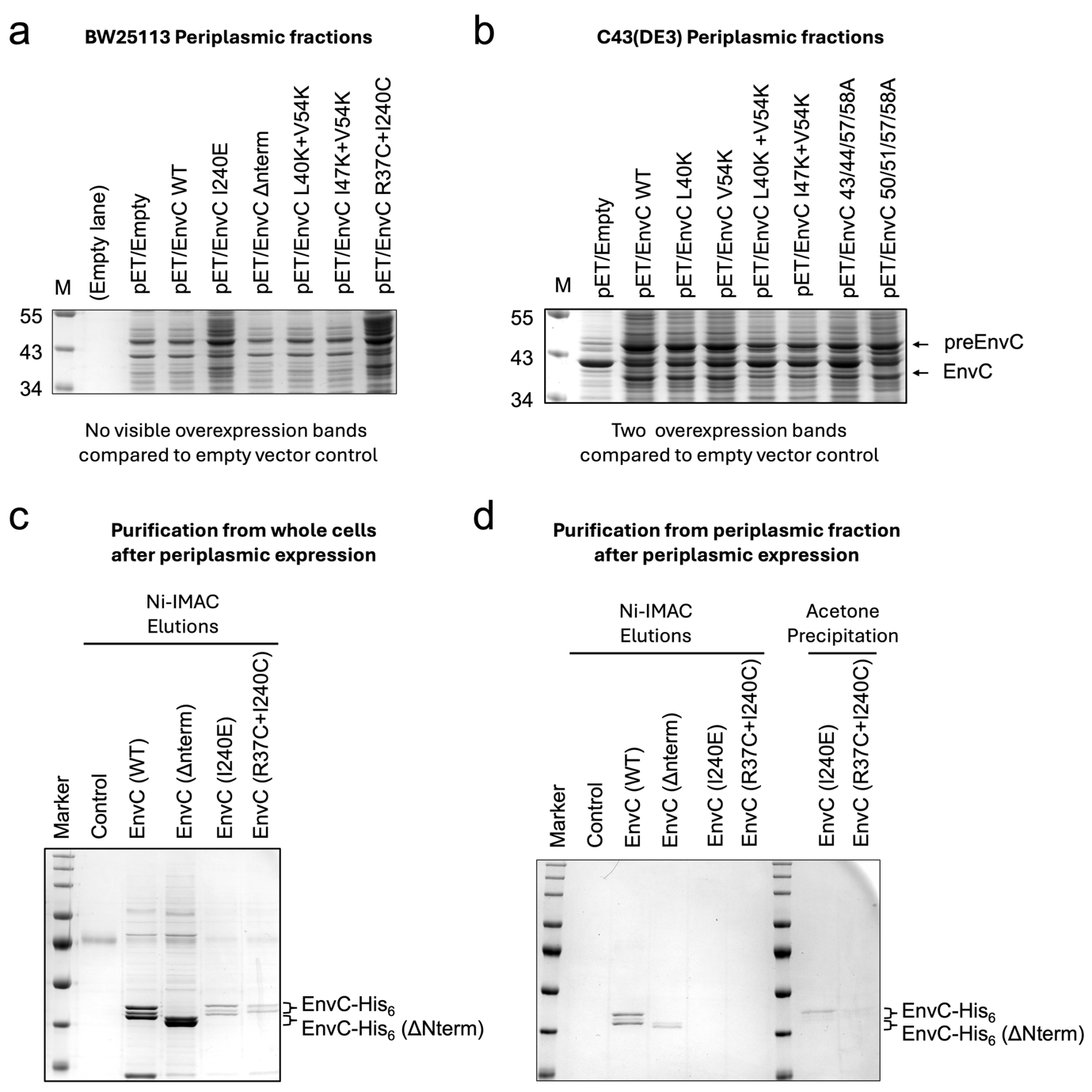


**Supplemental Figure S1: Periplasmic detection of plasmid-expressed EnvC variants to confirm expression.** (a) SDS-PAGE gel resolving proteins from the periplasmic fraction of *E. coli* BW25113. No visible EnvC overexpression was observed for any variant. Visible expression was not expected in BW25113 as it lacks the DE3 lysogen needed for high-level protein expression. (b) SDS-PAGE gel resolving proteins from the periplasmic fraction of *E. coli* C43(DE3). Additional bands corresponding to pre-EnvC (expected molecular weight 45,324 Da) and mature EnvC lacking its signal sequence (molecular weight 42,925 Da) are seen for all variants. (c) Purification of periplasm-expressed EnvC variants (with C-terminal His-tag) from whole cells. Ni-IMAC indicates Ni-Immobilised Metal Affinity Chromatography. ‘Control’ indicates cells grown with an empty vector and do not produce a His-tagged protein. (d) Purification of periplasm-expressed EnvC variants (with C-terminal His-tag). Both wild type EnvC and the EnvC(ΔNterm) variant could be purified from the periplasmic fraction (or whole cells) after periplasmic expression. The control, wild type and EnvCΔNterm samples were loaded evenly – but purified I240E and R37C+I240C EnvC variants were only detectable after loading additional material (4-fold amount). Low recovery of His-tagged I240E and R37C+I240C is most likely because of substantial lysis and leakage from the periplasm. Acetone precipitation was used to concentrate purified EnvC I240E and R37C+I240C sufficiently to observe on the gel (right-most lanes).


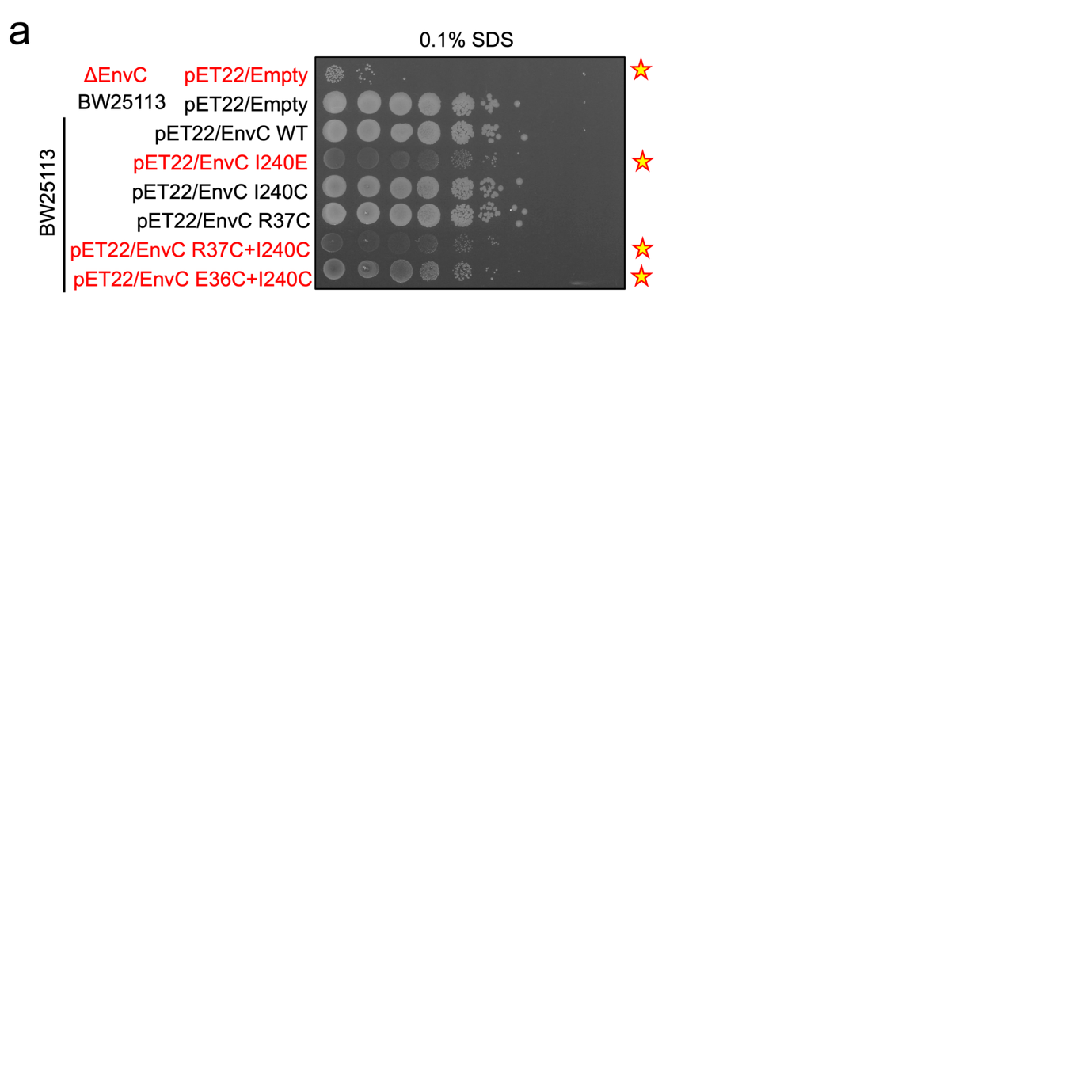


**Supplemental Figure S2: SDS sensitivity assay for *E. coli* BW25113 expressing EnvC variants.** A 10-fold serial dilution for each culture was spotted on LB agar containing 0.1 % SDS in series from left to right starting at OD_600_ 1. Stars indicate detergent sensitive strains. This figure is intended to complement main **Fig. 6b** which shows a similar experiment in the BW25113 ΔEnvC background.


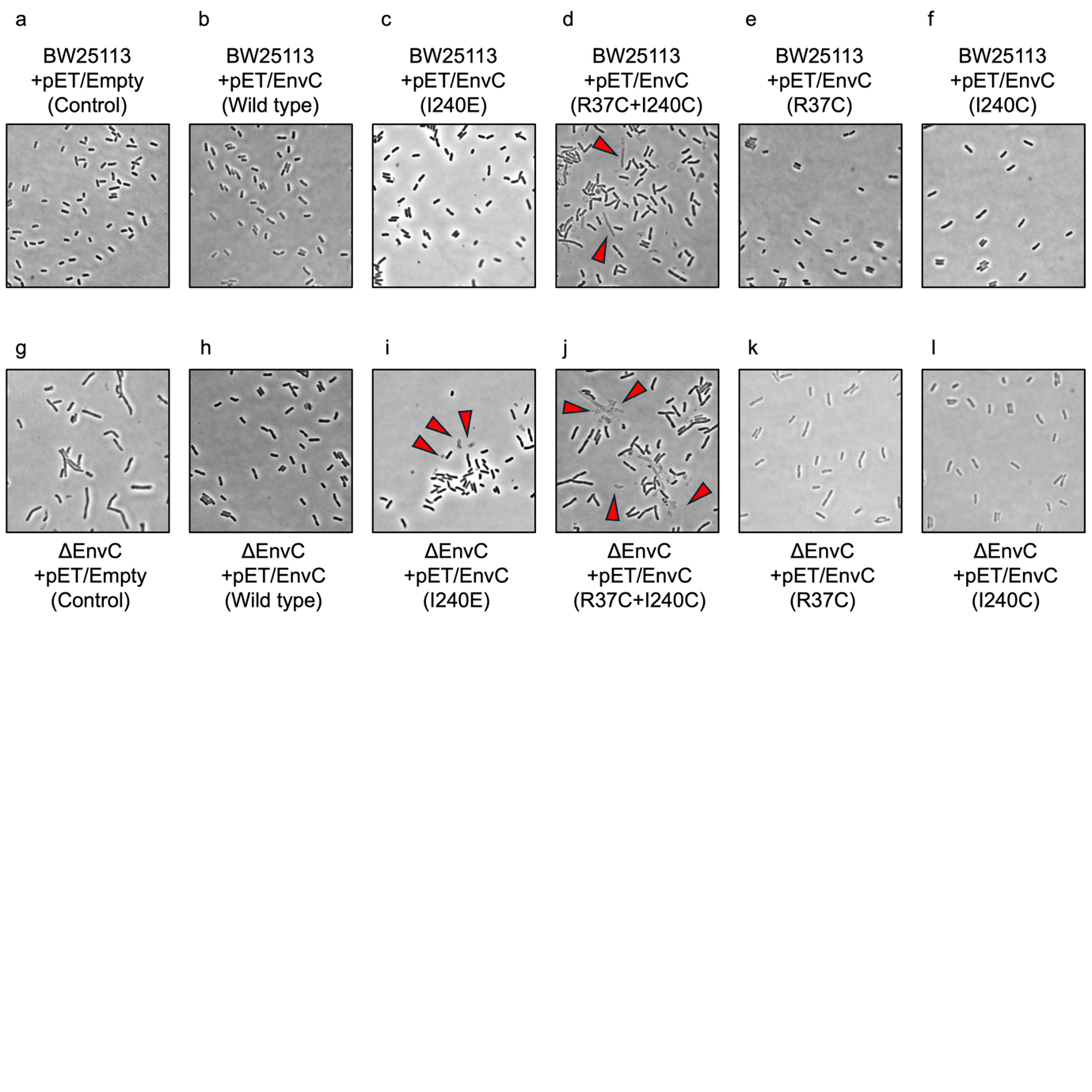


**Supplemental Figure S3: Phase contrast microscopy.** (a-l) Representative images for each strain are shown on a common scale. Arrows indicate examples of cellular debris.


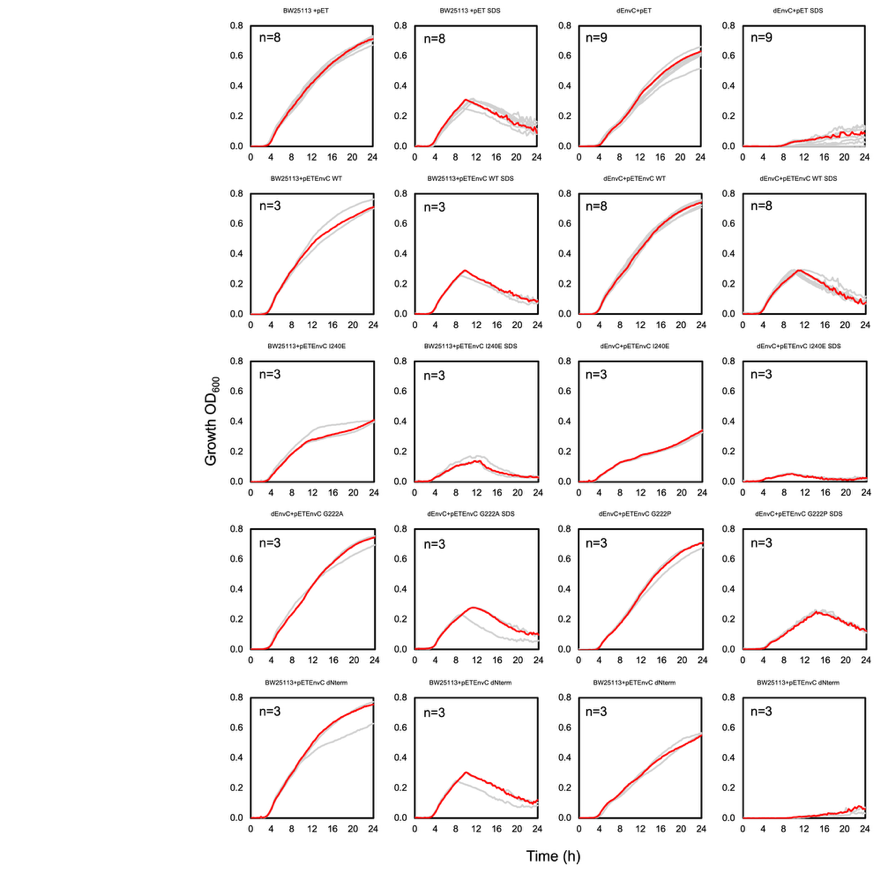

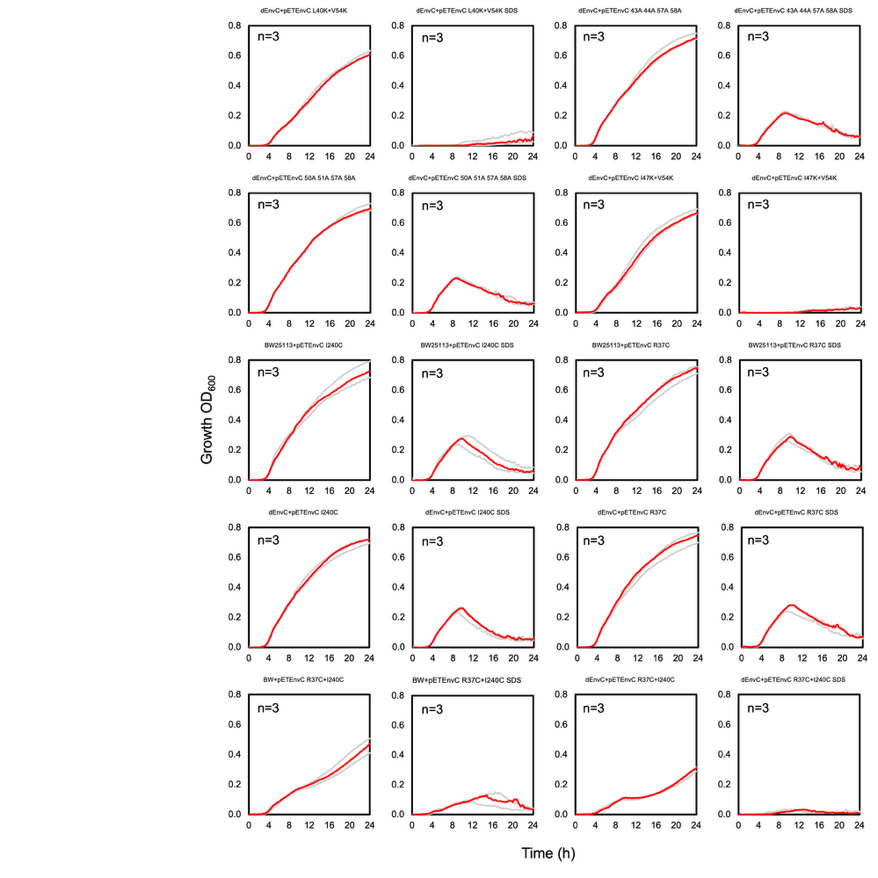


**Supplemental Figure S4: Growth curves repeat experiments.** The number of biological repeats (n) displayed is shown inset. Each biological repeat is the average of three technical repeats. A representative curve consisting of a single biological repeat was selected for use in the main figures; the selected curve is shown in *red* and the other biological repeats are shown in *grey*.
